# Irisin inhibits high glucose‐induced endothelial‐to‐mesenchymal transition and exerts a dose‐dependent bidirectional effect on diabetic cardiomyopathy

**DOI:** 10.1111/jcmm.13360

**Published:** 2017-10-23

**Authors:** Xue Liu, Haroon Mujahid, Bing Rong, Qing‐hua Lu, Wei Zhang, Peng Li, Na Li, Er‐shun Liang, Qi Wang, Dong‐qi Tang, Nai‐lin Li, Xiao‐ping Ji, Yu‐guo Chen, Yu‐xia Zhao, Ming‐xiang Zhang

**Affiliations:** ^1^ The Key Laboratory of Cardiovascular Remodeling and Function Research Chinese Ministry of Education and Chinese Ministry of Public Health Qilu Hospital of Shandong University Jinan Shandong China; ^2^ Department of Geriatrics Qilu Hospital of Shandong University Jinan Shandong China; ^3^ Department of Cardiology Second Hospital of Shandong University Jinan Shandong China; ^4^ Department of Pharmacology College of Pharmacy Xinxiang Medical University Xinxiang China; ^5^ Department of Medicine‐Solna Clinical Pharma Pharmacology Karolinska University Hospital‐Solna Stockholm Sweden

**Keywords:** irisin, diabetic cardiomyopathy, myocardial dysfunction

## Abstract

Emerging evidence indicates that irisin provides beneficial effects in diabetes. However, whether irisin influences the development of diabetic cardiomyopathy (DCM) remains unclear. Therefore, we investigated the potential role and mechanism of action of irisin in diabetes‐induced myocardial dysfunction in mice. Type 1 diabetes was induced in mice by injecting streptozotocin, and the diabetic mice were administered recombinant r‐irisin (low or high dose: 0.5 or 1.5 μg/g body weight/day, I.P.) or PBS for 16 weeks. Irisin treatment did not alter blood glucose levels in the diabetic mice. However, the results of echocardiographical and histopathological assays indicated that low‐dose irisin treatment alleviated cardiac fibrosis and left ventricular function in the diabetic mice, whereas high‐dose irisin failed to mitigate the ventricular function impairment and increased collagen deposition. The potential mechanism underlying the effect of low‐dose irisin involved irisin‐mediated inhibition of high glucose‐induced endothelial‐to‐mesenchymal transition (EndMT); conversely, high‐dose irisin treatment enhanced high glucose‐induced MMP expression by stimulating MAPK (p38 and ERK) signalling and cardiac fibroblast proliferation and migration. Low**‐**dose irisin alleviated DCM development by inhibiting high glucose‐induced EndMT. By contrast, high‐dose irisin disrupted normal MMP expression and induced cardiac fibroblast proliferation and migration, which results in excess collagen deposition. Thus, irisin can inhibit high glucose‐induced EndMT and exert a dose‐dependent bidirectional effect on DCM.

## Introduction

Diabetes mellitus is recognized to increase the risk of cardiomyopathy independently of coronary artery disease, hypertension and atherosclerosis 1. In DCM, which increases morbidity and mortality risk in diabetic patients, the pathological process presents myocardial diastolic and systolic dysfunction, left ventricular (LV) hypertrophy, and fibrosis 1, 2. Numerous complex molecular mechanisms have been proposed to investigate the structural and functional alterations in the diabetic heart, including increased perivascular and intermyofibrillar fibrosis and myocardial cell death in human diabetes 3, 4. However, the pathogenesis involved in the development and progression of cardiac dysfunction in diabetes remains only partially understood.

The mechanisms responsible for the myocardial fibrosis underlying the accumulation of extracellular matrix (ECM) proteins (particularly collagen types I and III) include excess collagen production, reduced degradation and chemical modification of ECM proteins 5. Cardiac fibroblasts (CFs) are the primary producers of the majority of collagen and other ECM proteins, and thus, CFs play a pivotal role in the pathogenesis of fibrosis in the diabetic heart. Recently, endothelial‐to‐mesenchymal transition (EndMT) has been suggested to represent a source of CFs: fibroblast‐like cells, derived from endothelial cells through EndMT, contribute to the pathogenesis of cardiac fibrosis 6. Furthermore, signalling molecules such as transforming growth factor‐β(TGF‐β) regulate this EndMT process by suppressing the expression of endothelial markers, and mitogen‐activated protein kinase (MAPK) signalling also participates in the development of DCM, with the inhibition of MAPK signalling attenuating DCM‐associated alterations 7, 8. Previous work has shown that TGF‐β is a potent mediator of cardiac fibrosis and that Smad proteins function as critical intracellular effectors in the TGF‐β signalling pathway: the overexpression of Smad7, an inhibitor of TGF‐β signalling, results in marked suppression of TGF‐β‐induced Smad2 activation and the prevention of collagen synthesis 9. The inhibitory binding of Smad7 also blocks the recruitment of Smad2 and Smad3 and suppresses TGF‐β signalling in cancer 10. Lastly, matrix metalloproteinases (MMPs), which constitute a major group of extracellular enzymes, play essential roles in ECM remodelling, and MMP activation leads to the degradation of the physiological collagen scaffold of the myocardium 11.

Irisin, a hormone that is formed from the precursor FNDC5 (fibronectin type III domain containing 5) and secreted into blood, is detected exclusively in skeletal muscle, heart and brain 12, 13; FNDC5 expression is stimulated by the PGC1α (PPARγcoactivator‐1α) that is induced by exercise 14. Irisin activates the browning of white fat and the expenditure of energy together with increased expression uncoupling protein 1 by stimulating the p38 and extracellular‐regulated protein kinase (ERK) signalling pathways, and thus, irisin ameliorates diet‐induced obesity and insulin resistance *in vivo* 15. Irisin serum levels were reported to be associated with an increased cardio metabolic risk 16, and the circulating level of irisin was found to be higher in patients with type 1 diabetes mellitus (T1DM) than in healthy people and notably lower in younger patients with myocardial infarction (MI) 17, 18; MI has been shown to be associated with a decrease in serum irisin levels 19. Moreover, diminished FNDC5 and PGC1α expression might underlie the reduced aerobic performance in heart failure patients 20.

The aforementioned findings related to irisin serum levels under certain pathophysiological conditions suggest that irisin could represent a novel therapeutic target in metabolic diseases. Here, to determine whether irisin influences cardiac fibrosis in diabetes, we investigated the precise effect of irisin on high glucose (HG)‐induced EndMT *in vitro* and the function of irisin in streptozotocin (STZ)‐induced DCM *in vivo*.

## Materials and methods

### Expression and purification of human Irisin

Human recombinant irisin (r‐irisin) was expressed and purified as previously described^15^. Briefly, human irisin cDNA (360 bp), designed and synthesized by Life Technologies, was cloned into the EcoRI/XbaI sites of the plasmid pPICZaA. According to the protocol provided with the PichiaEasyComp Transformation Kit (Invitrogen, Carlsbad, CA, USA), linearized pPICZaAirisin plasmid was used to transform *Pichia pastoris* X‐33, following which the yeast cells were cultured and r‐irisin expression was induced and r‐irisin protein in the culture supernatant was purified as described in a previous study (15).

### Animals model and treatments

C57/BL6J wild‐type mice (25–30 g) were purchased from the animal centre of Shandong University (Jinan, China). Diabetes was induced over 8 weeks through five consecutive days of intraperitoneal (I.P.) injection of each mouse with STZ (Sigma‐Aldrich, St. Louis, MO, USA), at the dose of 60 mg/kg body weight in 0.1 ml of citrate buffer (pH 4.5). After 5 days, blood glucose levels were randomly measured using an Accu‐Check Active glucometer (Roche, Basel, Switzerland), and the mice with glucose concentrations of >16 mmol/l were selected as the experimental diabetic model animals for the study. After 8 weeks, mice were divided randomly into four groups (*n *=* *6) and treated with r‐irisin (0.5 or 1.5 μg/g body weight/day, I.P.) (15) or saline (at the same volume) for 16 weeks, after which the mice were anaesthetized and then killed. All the experimental procedures conformed to the Guide for Shandong University and the Care and Use of Laboratory Animals published by the US National Institutes of Health.

### Blood analysis

The levels total cholesterol, triglycerides, HDL, LDL and FBG were measured using a Bayer1650 blood chemistry analyser (Bayer, Tarrytown, NY, USA).

### Echocardiography

The imaging systemVevo770 (Visual Sonics, Toronto, ON, Canada) was used for measuring cardiac diameter and function. To measure LV ejection fraction (LVEF), we performed 2D echocardiography, M‐mode echocardiography (for the thickness of the septum and posterior wall), pulsed‐wave Doppler echocardiography (E/A) and tissue Doppler imaging (for LV diastolic dysfunction). We also measured fractional shortening (FS), the ratio of early to late mitral inflow velocity (E/A), the ratio of diastolic mitral annulus velocities (E′/A′) and LV end‐diastolic dimension (LVEDd).

### Western blotting

Proteins were extracted from dissected mouse hearts or cultured cells, and equal amounts of protein were separated using 10% sodium dodecyl sulphate‐polyacrylamide gel electrophoresis (SDS‐PAGE) and then transferred to polyvinylidene fluoride membranes. The membranes were blocked for 2 hrs with 5% non‐fat milk, washed for 5 min. in Tris‐buffered saline containing 0.1% Tween‐20 (TBS‐T) and then incubated overnight at 4°C with primary antibodies; the antibodies used were against collagens I and III, VE‐cadherin, CD31, α‐smooth muscle actin (α‐SMA), vimentin, fibroblast‐specific protein (FSP)‐1, MMP‐2 and MMP‐9 (Abcam, Cambridge, MA, USA); and p38 MAPK, phosphor (p)‐p38 MAPK, ERK1/2 and p‐ERK1/2 (Cell Signaling Technology, Beverly, MA, USA). The membranes were washed thrice for 10 min. with TBS‐T, incubated with horseradish peroxidase‐conjugated secondary antibodies (1:10,000, 1 hr, room temperature), washed thrice more for 15 min. with TBS‐T and then incubated with enhanced chemiluminescence substrate to visualize the immune‐reactive protein bands.

### Histology and immunohistochemical staining

Heart tissue samples were fixed in 4% formalin, embedded in paraffin, sectioned (4 μm) and then used for haematoxylin and eosin (H&E) staining and immunohistochemical staining. The widths of the cardiomyocytes in the tissue samples were measured by examining the H&E‐stained sections. In other cases, sections were labelled with primary antibodies against mouse collagens I and III (Abcam) and then with goat anti‐rabbit secondary antibodies to examine these specific collagens, or stained with Picrosirius red and Masson's trichrome to visualize total collagen and thus determine the proportion of the areas positive for collagen deposition. All histopathological sections were analysed using Image‐Pro Plus 6.0.

### Cell culture

The stable, human umbilical vein endothelial cells (HUVECs) were purchased from American Type Culture Collection (ATCC, Manassas, VA, USA). HUVECs were incubated with 5 or 33 mmol/l d‐glucose medium. Before glucose treatment, the HUVECs were incubated with 20 or 40 nmol/l r‐irisin for 8 hrs. CFs were isolated from ventricular tissues of 1‐ to 3‐day‐old neonatal mice. The atria and left ventricle were removed and minced separately, and the minced sections were washed twice with DMEM. The sections were suspended in 5 ml of collagenase type II in DMEM and incubated at 37°C for 1 hr. CFs were identified by immunofluorescence and cell morphological analysis as described previously 21. CFs were incubated with r‐irisin at different concentrations and then exposed to normal glucose (NG; 5 mmol/l) or HG (33 mmol/l) for 24 or 48 hrs.

### Immunofluorescence analysis

Cultured HUVECs seeded in 24‐well plates were fixed in 4% paraformaldehyde for 15 min., washed thrice for 10 min. each in PBS and then incubated (overnight, 4°C) with two primary antibodies; the antibodies used (from Abcam, Cambridge, MA, UK) were mouse anti‐CD31 (1:200), rabbit anti‐FSP‐1 (1:100), mouse anti‐α‐SMA (1:200) and rabbit anti‐VE‐cadherin (1:400). After washing thrice for 10 min. each in PBS, cells were incubated with a mixture of two secondary antibodies for 1 hr at room temperature; the secondary antibodies were goat anti‐rabbit Alexa Fluor 549 (1:200) and goat antimouse Alexa Fluor 488 (1:200). Nuclei were counterstained with 4′‐6‐diamidino‐2‐phenylindole (DAPI; Invitrogen). The immunofluorescent staining was analysed using laser‐scanning confocal microscopy (LSM710, Carl Zeiss, Germany), and the cells showing or not showing irisin effects were photographed.

### Flow cytometry

Cardiac fibroblasts were cultured in 6‐well plates for 48 hrs in the presence or absence of 80 nmol/l irisin and exposed to NG (5.5 mmol/l) or HG (25 mmol/l), after which the cell amounts were determined. Cells were collected and washed twice with PBS, fixed with 1% paraformaldehyde (methanol‐free) and stored in 70% ethanol at −20°C overnight, and then, 500 μl of propidium iodide (PI) (BD, CA) was added to each sample and the samples were incubated for 15 min. in the dark at room temperature. Cell cycle was analysed by performing flow cytometry on a BD AccuriC6 flow cytometer.

### EDU Assay

The EDU assay was performed using a 5‐ethynyl‐2′‐deoxyuridine labelling detection kit (EdU, Ribobio, CN). Serum‐starved CFs were seeded in 96‐well plates, incubated with 50 μmol/l EdU for 12 hrs, fixed in 4% paraformaldehyde for 30 min. and then treated with 2 mg/ml glycine for 5 min. After washing, 100 μl of 0.5% Triton X‐100 was added per well and incubated at 37°C for 10 min., and then, the wells were washed thrice with PBS and stained with 100 μl of 1× Apollo^®^ reaction cocktail for 30 min. at room temperature. Subsequently, the DNA in the CFs was stained with Hoechst 33342 for 30 min. Images were obtained using a laser‐scanning confocal microscope (LSM710; Carl Zeiss). The Edu‐positive cell index is the ratio of the number of positive cells to the total number of cells.

### Migration assay

Migration assays were performed using Transwell cell chambers (8 μm pore size; Corning Incorporated, Corning, NY, USA) in 24‐well dishes. Briefly, CFs (10^4^ cells in 200 μl of starvation medium) were adhered to the upper Transwell chamber for 10 hrs at 37°C, and 500 μl of DMEM containing 10% FBS with or without 20 nmol/l irisin was loaded into the lower chamber. CFs were incubated for 10 hrs at 37°C in 5% CO_2_ and recovered with 4% formaldehyde for 5 min., after which the membrane was stained with 0.1% crystal violet for 30 min. Lastly, the migrated cells in five randomly selected (200×) fields under a microscope were counted.

### Statistical analysis

Results are presented as means ± S.D. Groups were compared using one‐way anova analysis; Graph Pad Prism 6 software (Graph Pad software Inc., USA) was used for the analyses. *P *< 0.05 was considered significant.

## Results

### Dose‐dependent opposing effects of r‐Irisin treatment on diabetes‐induced myocardial remodelling

**Figure 1:**
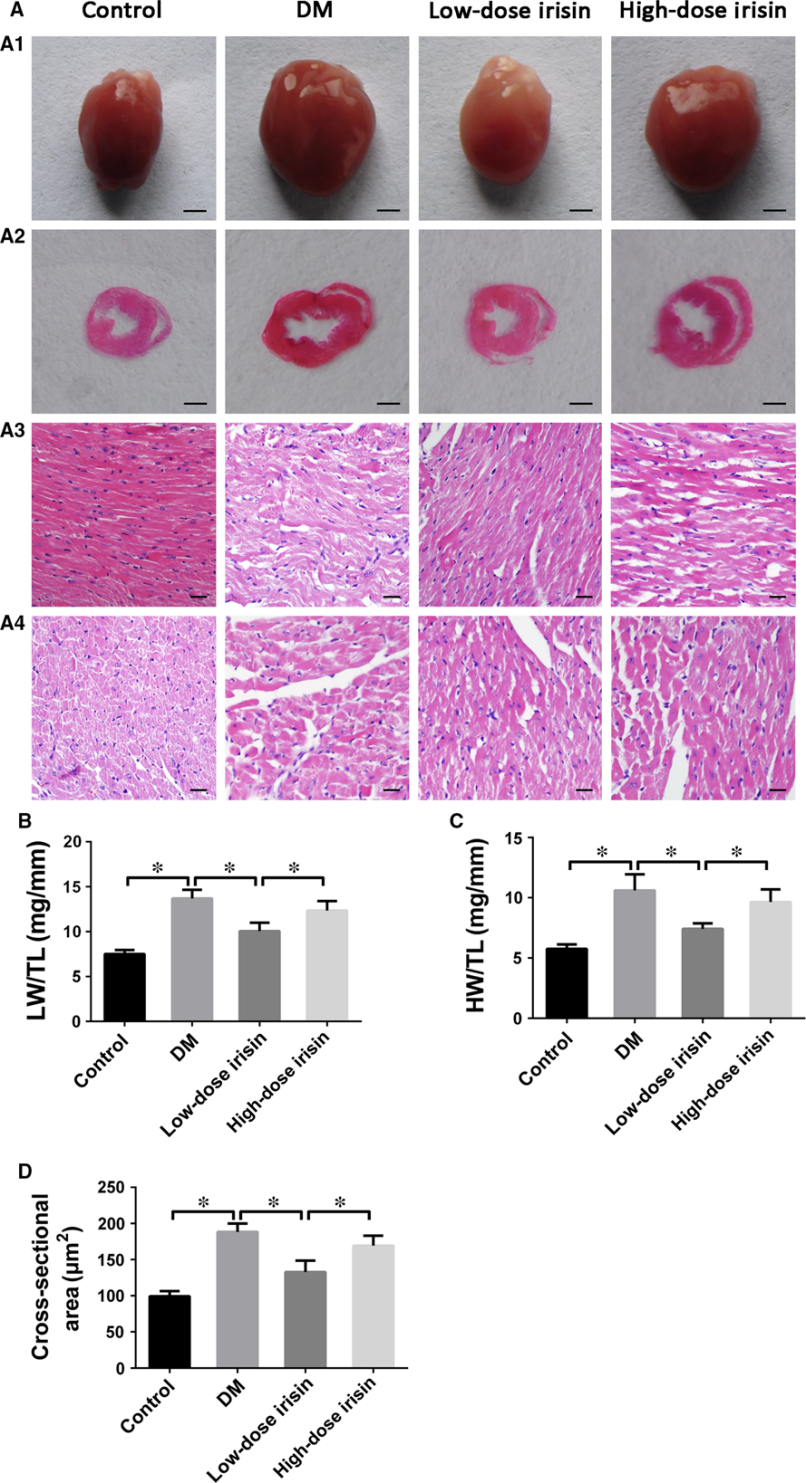
Effect of r‐irisin on myocardial remodelling in mice with STZ‐induced diabetes. A1: Heart size comparison (scale bar: 3 mm). A2: Representative histologic cross‐sectional anatomy at the papillary muscle level (scale bar: 3 mm). A3: Representative haematoxylin and eosin staining (H&E) of left ventricular (LV) longitudinal sections (scale bar: 20 μm). A4: Representative H&E staining of LV transverse sections (scale bar: 20 μm). (**B**) Lung weight/tibial length (LW/TL) ratios. (**C**) Heart weight/tibial length (HW/TL) ratios. (**D**) Cross‐sectional area of cardiomyocytes quantified from H&E‐stained sections. Control: normal mice treated with PBS. DM: diabetic mice treated with PBS. Low‐dose Irisin: diabetic mice treated with low dose of r‐irisin. High‐dose Irisin: diabetic mice treated with high dose of r‐irisin. Results shown are means ± S.D.; *P < 0.05.

To investigate the effect of r‐irisin on diabetes‐induced myocardial remodelling, we treated normal and DM mice with daily injections of r‐irisin or saline at the same volume for 16 weeks (starting at 8 weeks after the establishment of diabetes). Blood glucose, body weight, and heart and lung weight were measured, and the ratios of heart weight and lung weight to tibial length were calculated. DM was associated with elevated blood glucose level (Table 1). The heart weight to tibial length and lung weight to tibial length ratios were also significantly higher in the DM group than in the control group, and, notably, both of these ratios were decreased after low‐dose r‐irisin treatment for 16 weeks (Fig. 1A–C). However, no difference was detected between the DM group and the high‐dose r‐irisin group. These results indicate that irisin exerts a dose‐dependent bidirectional effect on cardiac remodelling. Moreover, blood pressure was lower in both r‐irisin groups than in the control and DM groups, and the cardiomyocyte cross‐sectional area was lower in the low‐dose irisin group than in the DM and high‐dose irisin groups (Fig. 1D).

**Table 1:** Blood glucose

**Blood glucose (mmol/l)**	
Control	8.18 ± 0.24
DM	25.33 ± 3.61*
Low‐dose Irisin	24.37 ± 3.48
High‐dose Irisin	26.78 ± 2.88

Data were obtained at 24 weeks after diabetes induction. All groups, *n *=* *6 mice. Control: normal mice treated with PBS. DM: diabetic mice treated with PBS. Low‐dose Irisin: diabetic mice treated with r‐irisin at the dose of 0.5 μg/g body weight/day. High‐dose Irisin: diabetic mice treated with r‐irisin at 1.5 μg/g body weight/day. Results are presented as means ± S.D.

*P <* *0.05 *versus* Control.

### Distinct dosage‐dependent effects of r‐Irisin on diabetes‐induced myocardial dysfunction

**Figure 2:**
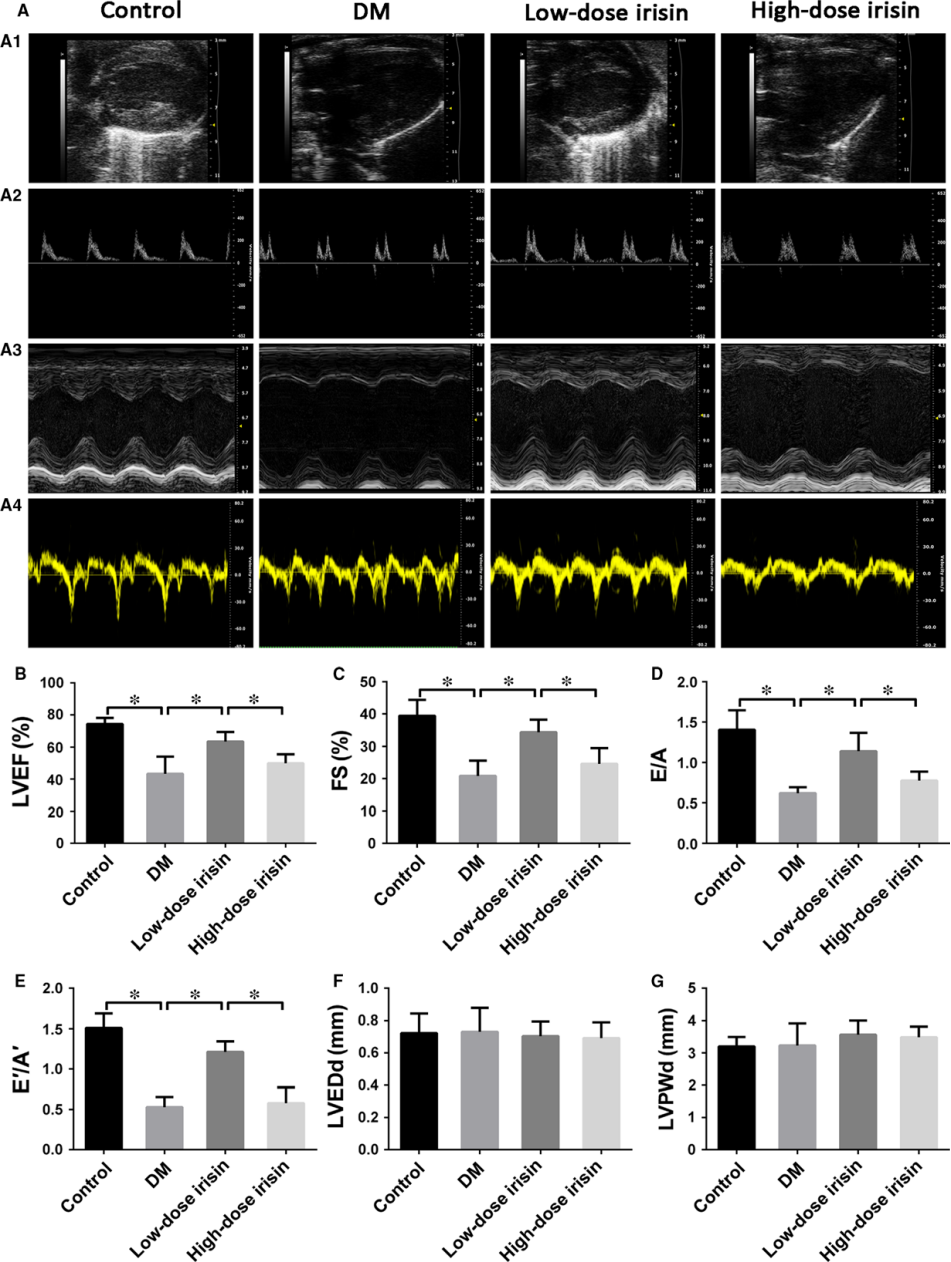
Effect of r‐irisin on diabetes‐induced myocardial dysfunction. A1: Representative 2D echocardiograms. A2: Representative M‐mode echocardiograms. A3: Representative pulsed‐wave Doppler echocardiograms of mitral inflow. A4: Representative tissue Doppler echocardiograms. (**B**) LV ejection fraction (LVEF). (**C**) Fractional shortening (FS). (**D**) Early to late mitral flow (E/A). (**E**): E′/A′. (**F**): LV end‐diastolic dimension (LVEDd). (**G**) LV posterior wall thickness at diastole (LVPWd). Control: normal mice treated with PBS. DM: diabetic mice treated with PBS. Low‐dose Irisin: diabetic mice treated with low dose of r‐irisin. High‐dose Irisin: diabetic mice treated with high dose of r‐irisin. Results are shown as means ± S.D.; *P < 0.05.

Cardiac function was assessed using echocardiography. At 12 weeks after the establishment of diabetes, cardiac functional features were markedly impaired (Fig. 2A), and myocardial dysfunction was clearly evident in the DM group as compared with the control group; this was indicated by the lower LVEF, FS, E/A ratio and E'/A' ratio in the DM group (Fig. 2B–E). The low‐dose r‐irisin group (at 16 weeks) exhibited improved cardiac functions as compared with the high‐dose irisin group, which showed no marked improvement relative to the DM group, as indicated by the LVEF, FS, E/A ratio and E'/A' ratio. The increased LV posterior wall thickness at diastole and LVEDd (Fig. 2F and G), which indicate ventricular remodelling, did not differ significantly between the groups. These results also showed the bidirectional effect of irisin on cardiomyocytes.

### Dose‐dependent bidirectional effect of r‐Irisin on diabetes‐induced myocardial fibrosis

**Figure 3:**
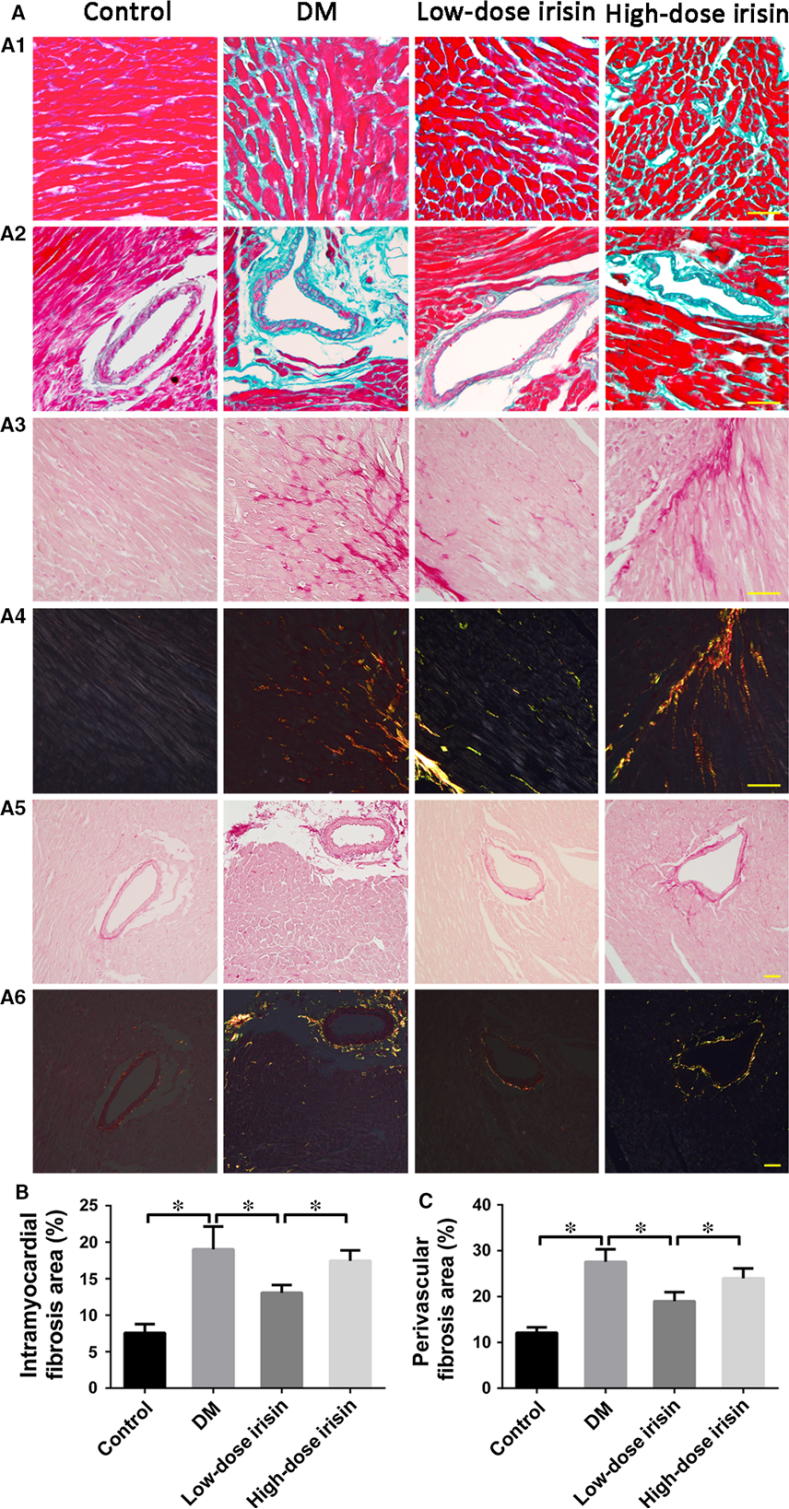
Effect of r‐irisin on diabetes‐induced myocardial fibrosis. A1‐A2: Representative Masson's trichrome staining of the myocardium (scale bar: 30 μm). A3‐A6: Representative Picrosirius red staining of the myocardium (scale bar: 50 μm). (**B**) Quantified intramyocardial fibrosis area. (**C**) Quantified perivascular fibrosis area. Control: normal mice treated with PBS. DM: diabetic mice treated with PBS. Low‐dose Irisin: diabetic mice treated with low dose of r‐irisin. High‐dose Irisin: diabetic mice treated with high dose of r‐irisin. Results are means ± S.D.; *P < 0.05.

Masson's trichrome and Picrosirius red staining revealed a substantial increase in collagen deposition in the DM group relative to the control group (Fig. 3). Treatment with low‐dose irisin, but not high‐dose irisin, resulted in a statistically significant decrease in collagen deposition as compared with the DM group; moreover, the intramuscular and perivascular fibrosis areas were significantly lower in the low‐dose irisin group than in the DM and high‐dose irisin groups (Fig. 3). Once again, no difference was detected between the DM and high‐dose irisin groups.

### Distinct effects of low‐ and high‐dose r‐Irisin treatment on collagen deposition in the diabetic myocardium

**Figure 4:**
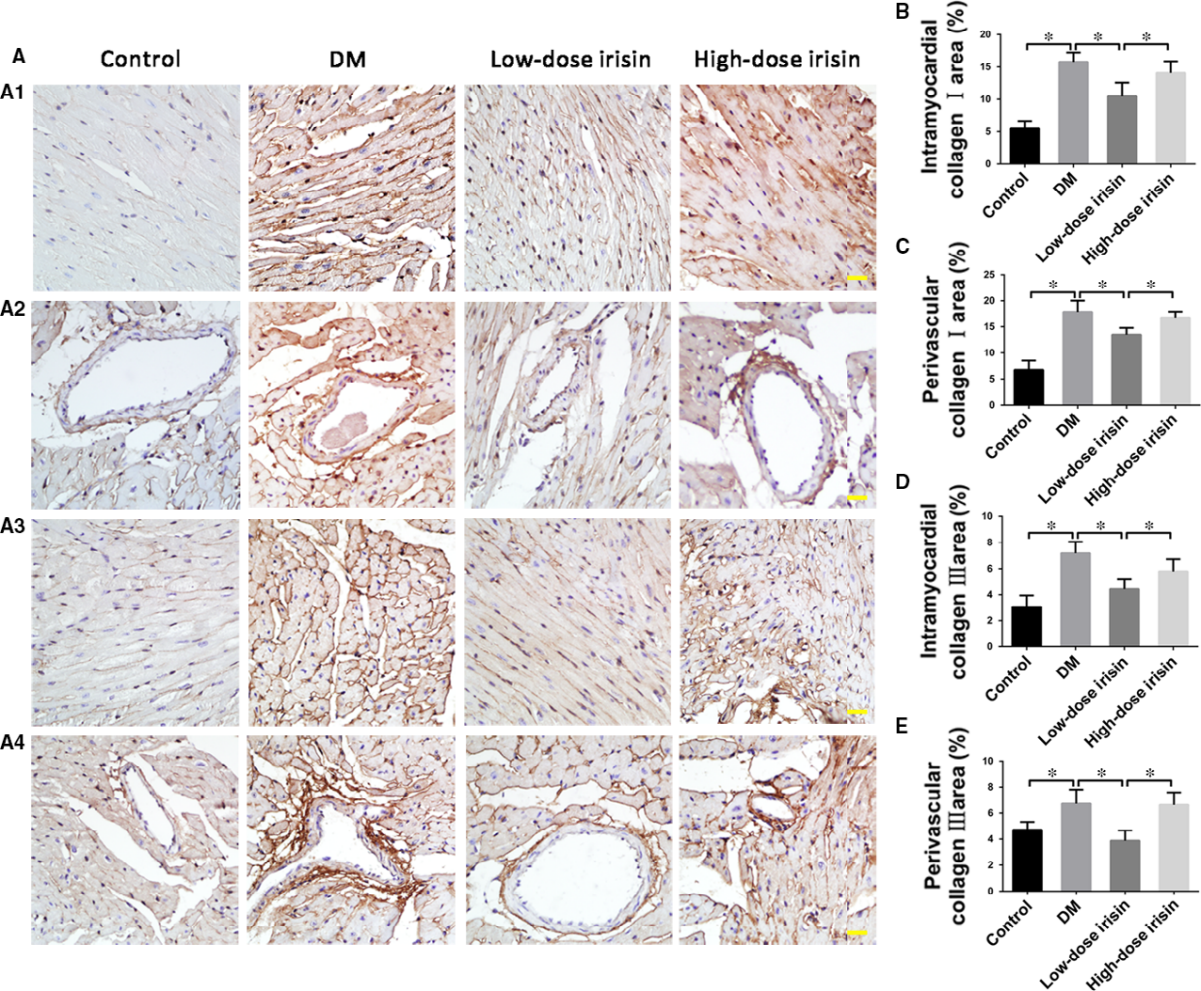
Effect of r‐irisin on collagen I and III levels in mice with STZ‐induced diabetes. (**A**) Representative immunohistochemical staining of collagens I (A1‐A2) and III (A3‐A4) (scale bar: 30 μm). (**B**–**C**) Quantification of intramyocardial (**B**) and perivascular (**C**) areas positive for collagen I. (**D–E**) Quantification of intramyocardial (**D**) and perivascular (**E**) areas positive for collagen III. Control: normal mice treated with PBS. DM: diabetic mice treated with PBS. Low‐dose Irisin: diabetic mice treated with low dose of r‐irisin. High‐dose Irisin: diabetic mice treated with high dose of r‐irisin. Results shown are means ± S.D.; *P < 0.05.

Immunohistochemical analysis was performed to examine intramyocardial and perivascular expression of collagens I and III in the LV tissue (Fig. 4). Expression of collagens I and III was significantly up‐regulated in the DM group relative to that in the control group, but low‐dose irisin treatment led to down‐regulation of the collagen expression as compared with level in the DM group (Fig. 4). Collagen expression did not differ markedly between the DM and high‐dose irisin groups.

### R‐Irisin treatment suppresses HG‐induced EndMT *in vivo* and *in vitro*

**Figure 5:**
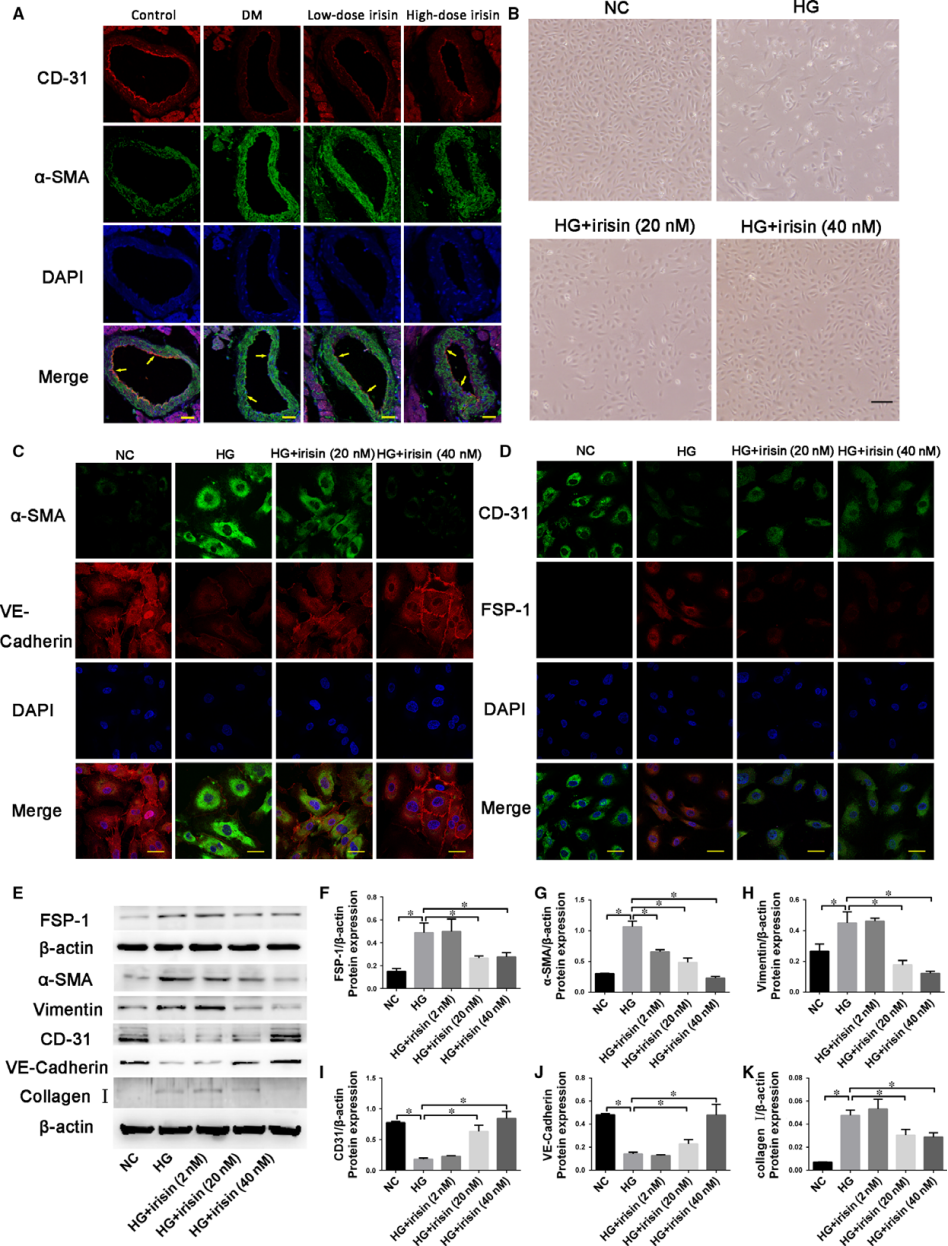
Effect of r‐irisin on high glucose (HG) induced EndMT *in vivo* and *in vitro*. (**A**) Double immunofluorescence staining of antibodies to CD31 (Red) with antibodies to α‐SMA (green) in coronary arterioles of all groups mouse. Colocalization of CD31 with α‐SMA expression in coronary arterioles is shown in yellow. DAPI (Blue) was used to stain nucleus. Scale bars = 30 μm. (**B**) Morphological change in HUVECs after HG exposure with or without r‐irisin treatment, which results in a fibroblast‐like spindle‐shaped form (scale bar: 100 μm). (**C**) Double immunofluorescence staining with antibodies against α‐SMA (green) and VE‐cadherin (red) (scale bar: 40 μm). (**D**) Double immunofluorescence staining with antibodies against CD31 (green) and S100A4/FSP‐1 (red) (scale bar: 40 μm). Nuclei were counterstained with DAPI (blue in **A**,** C** and **D**). E‐K: Western blotting analysis (**E**) and quantification of the protein expression of S100A4/FSP‐1(**F**), α‐SMA(**G**), vimentin(**H**), CD31(**I**), VE‐cadherin(**J**) and collagen I(**K**). NG (normal glucose): 5 mmol/l glucose. HG: 33 mmol/l glucose. HG+Irisin (2 nmol/l): 33 mmol/l glucose + 2 nmol/l r‐irisin. HG+Irisin (20 nmol/l): 33 mmol/l glucose + 20 nmol/l r‐irisin. HG+Irisin (40 nmol/l): 33 mmol/l glucose + 40 nmol/l r‐irisin. Results are shown as means ± S.D.; *P < 0.05.

We performed double immunofluorescence staining with antibodies to CD31 and α‐SMA *in vivo*. We observed colocalization of CD31 and α‐SMA expression in the endothelial layer of interstitial tissue and microcapillary vessels. The *in vivo* expression of the endothelial marker CD31 was lower in the DM group than in the low‐ and high‐dose irisin treatment groups, both of which exhibited strong CD31 expression; conversely, the expression of the mesenchymal marker α‐SMA was higher in the DM group than in the low‐ and high‐dose irisin groups (Fig. 5A). The same effect was also observed *in vitro*. Following treatment of HUVECs with HG for 5 days with or without exposure to different concentrations of irisin, the cells presented typical rounded or cobblestone shapes; notably, irisin treatment inhibited the HG‐induced transformation of the cell shape from cobblestone‐like to spindle‐like (Fig. 5B). Furthermore, the results of immunofluorescence analysis and Western blotting (Fig. 5C–K) indicated that HG treatment caused a marked increase (relative to control) in the levels of the mesenchymal markers FSP‐1, α‐SMA, collagen I, and vimentin and a reduction in the levels of the endothelial markers CD31 and VE‐cadherin, and that irisin treatment dose dependently decreased FSP‐1, α‐SMA, vimentin and collagen I levels and increased CD31 and VE‐cadherin levels.

### Effect of r‐irisin on CF proliferation, migration, and collagen secretion

**Figure 6:**
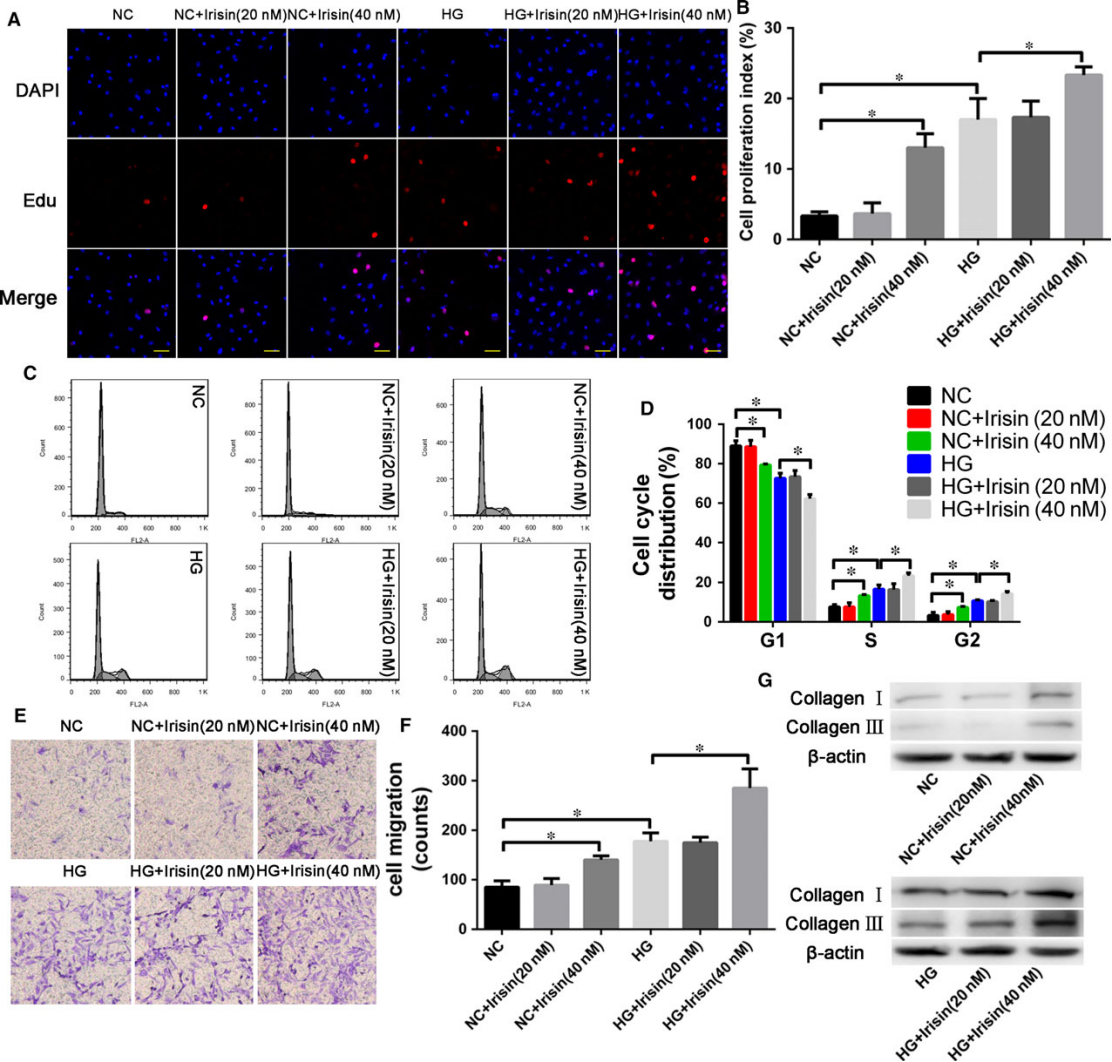
Effect of r‐irisin on cardiac fibroblast (CF) proliferation and migration. (**A**) EDU assay of HG/r‐irisin induced CF proliferation (scale bar: 40 μm). (**B**) Percentages of EDU‐positive cells. (**C**) Flow cytometry performed with propidium iodide (PI) staining to analyse CF proliferation. (**D**) Analysis of cell cycle distribution. (**E**) CFs were cultured in Transwells (pore size, 8 μm) for 12 hrs, and then, the CFs on the external surface of the Transwells were stained with crystal violet and photographed under a microscope. (**F**) Quantification of CF chemotaxis and migration towards HG/r‐irisin. (**G**) Western blotting analysis of the expression of collagens I and III in cells treated with different doses of irisin. NG: 5 mmol/l glucose. NG+Irisin (20 nmol/l): 5 mmol/l glucose + 20 nmol/l r‐irisin. NG+Irisin (40 nmol/l): 5 mmol/l glucose + 40 nmol/l r‐irisin. HG: 33 mmol/l glucose. HG+Irisin (20 nmol/l): 33 mmol/l glucose + 20 nmol/l r‐irisin. HG+Irisin (40 nmol/l): 33 mmol/l glucose + 40 nmol/l r‐irisin. Results are shown as means ± S.D.; *P < 0.05.

We examined how r‐irisin affects CF proliferation using the EDU assay; here, CFs were incubated with r‐irisin at various doses with or without HG exposure for 48 hrs. HG exposure promoted CF proliferation relative to control, and treatment with high dose of r‐irisin significantly enhanced the proliferation‐promoting effect of HG on CFs (Fig. 6A and B). To determine whether this effect of r‐irisin on cell growth was mediated by cell cycle arrest, we used flow cytometry to analyse the CF cell cycle distribution (Fig. 6C and D): HG‐induced CF proliferation by more strongly promoting, relative to NG, the G1‐to‐S‐phase transition of cells, and under both NG and HG exposure, 48‐hrs irisin treatment (40 nmol/l) led to a reduction in the proportion of cells at G1 and an increase at G2 and S phases. The specific proportions of CFs at G1 were 88.53%, 89.74%, 80.21%, 72.33%, 75.5% and 62.00% in the groups NG, NG+Irisin (20 nmol/l), NG+Irisin (40 nmol/l), HG, HG+Irisin (20 nmol/l) and HG+Irisin (40 nmol/l), respectively; moreover, the fraction of cells at G2 was increased to 10.00% and 16.67% in NG+Irisin (40 nmol/l) and HG+Irisin (40 nmol/l) groups from 4.00% and 11.00% in the NG and HG controls, respectively (Fig. 6C and D). The results of Transwell assays showed that high‐dose but not low‐dose r‐irisin treatment caused an increase in both NG‐ and HG‐induced CF migration (Fig. 6E and F), and Western blotting analysis revealed that high‐dose r‐irisin treatment caused increased collagen secretion by CFs as compared with the secretion level after low‐dose r‐irisin treatment, in which case the level was not markedly different from control (Fig. 6G).

### TGF‐β/Smad signalling may involve in mediating the irisin effect on HG‐induced EndMT

**Figure 7:**
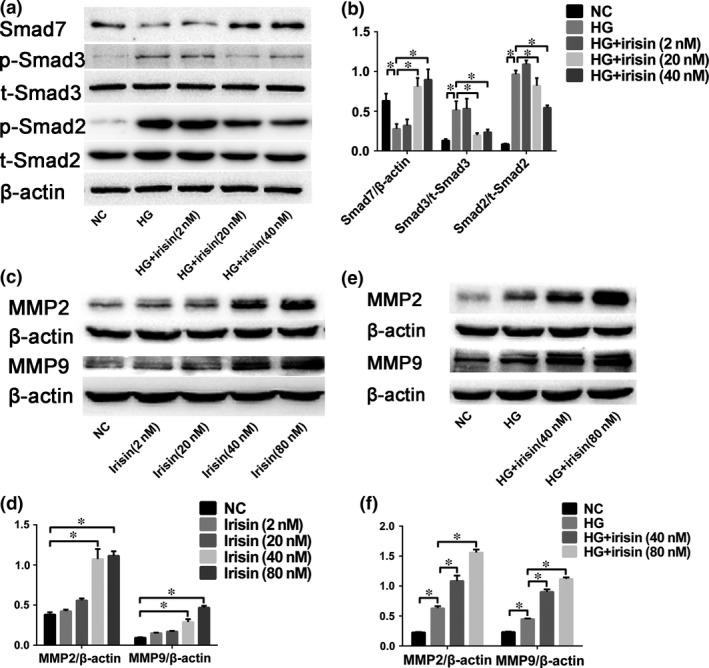
Effect of r‐irisin on Smad expression/phosphorylation in HUVECs and matrix metalloproteinase (MMP) expression in CFs. (**A–B**) Western blotting analysis (**A**) and quantification (**B**) of Smad7, p‐Smad2/t‐Smad2 and p‐Smad3/t‐Smad3 levels in HUVECs after HG exposure with or without r‐irisin treatment. (**C**) CFs were stimulated with 0, 2, 20, 40 or 80 nmol/l r‐irisin in NG medium, and then, MMP‐2 and MMP‐9 expression was analysed through Western blotting. (**D**) Quantification of MMP‐2 and MMP‐9 expression. (**E–F**) Western blotting analysis (**E**) and quantification (**F**) of MMP‐2 and MMP‐9 expression in CFs after NG exposure or after HG exposure with or without r‐irisin at 0, 40 or 80 nmol/l. NG: 5 mmol/l glucose. HG: 33 mmol/l glucose. HG+Irisin (2 nmol/l): 33 mmol/l glucose + 2 nmol/l r‐irisin. HG+Irisin (20 nmol/l): 33 mmol/l glucose + 20 nmol/l r‐irisin. HG+Irisin (40 nmol/l): 33 mmol/l glucose + 40 nmol/l r‐irisin. HG+Irisin (80 nmol/l): 33 mmol/l glucose + 80 nmol/l r‐irisin. Results are mean ± S.D.; *P < 0.05.

To identify the signalling proteins involved in mediating the irisin effect, we examined the level of TGF‐β/Smad signalling *in vitro*. In HG‐treated HUVECs, the levels of phosphorylated Smad2 and Smad3 were increased and Smad7 expression was decreased as compared with the corresponding levels in untreated cells. Irisin dose dependently decreased the levels of phosphorylated Smad2 and Smad3 and increased Smad7 expression (Fig. 7A and B).

### R‐irisin modulates the expression of ECM‐related proteins in CFs

We examined MMP‐2 and MMP‐9 expression in CFs to assess the effect of irisin on ECM homeostasis. MMP‐2 and MMP‐9 protein levels were increased relative to those in the NG group in a dose‐dependent manner after r‐irisin treatment, although low‐dose r‐irisin (2 or 20 nmol/l) did not markedly affect the expression (Fig. 7C and D). Moreover, r‐irisin at high doses (40 or 80 nmol/l) significantly enhanced HG‐induced increase in MMP‐2 and MMP‐9 protein expression (Fig. 7E and F).

### R‐Irisin intensifies HG‐induced p38MAPK and ERK activation in CFs

**Figure 8:**
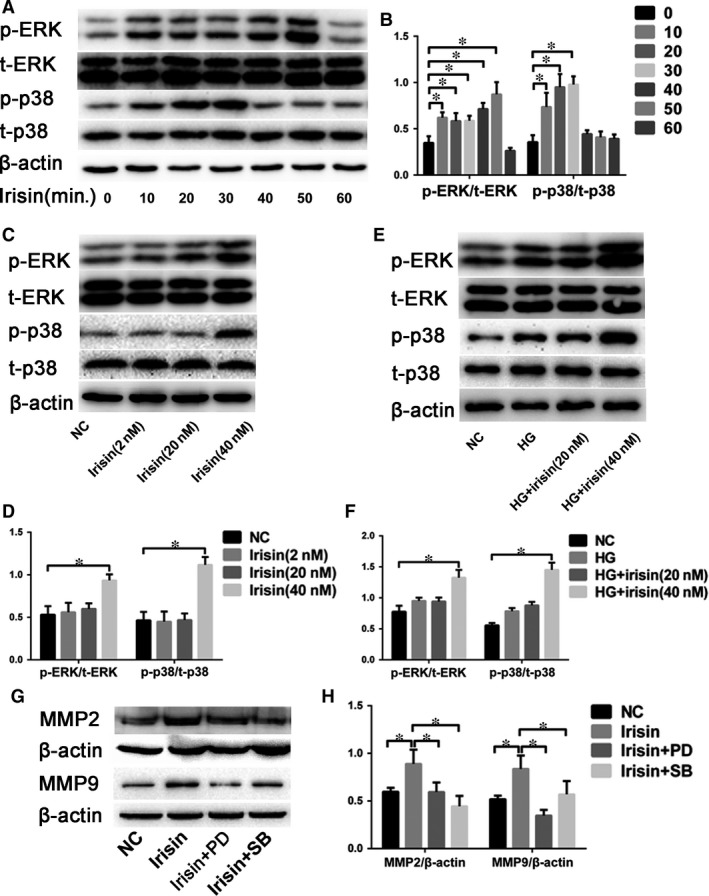
R‐irisin intensifies HG‐induced activation of p38MAPK and ERK in CFs. (**A‐B**) Western blotting analysis (**A**) and quanti fication (**B**) of p‐p38/t‐p38 and p‐ERK/t‐ERK levels in CFs at the indicated time‐points. (**C–D**) Western blotting analysis (**C**) and quantification (**D**) of p‐p38/t‐p38 and p‐ERK/t‐ERK levels in CFs after treatment with r‐irisin at 0, 2, 20, 40 nmol/l in NG medium. (**E–F**) Western blotting analy sis (**E**) and quantification (**F**) of p‐p38/t‐p38 and p‐ERK/t‐ERK levels in CFs after NG exposure or after HG exposure with r‐irisin treatment at 0, 20 or 40 nmol/l. G‐H: Western blotting analysis (**G**) and quantification (**H**) of MMP‐2 and MMP‐9 expression in CFs after NG or HG/r‐irisin treatment with PD or SB. NG: 5 mmol/l glucose. HG: 33 mmol/l glucose. HG+Irisin (20 nmol/l): 33 mmol/l glucose + 20 nmol/l r‐irisin.HG+Irisin (40 nmol/l): 33 mmol/l glucose + 40 nmol/l r‐irisin. Irisin+PD: 40 nmol/l r‐irisin with ERK inhi bitor PD98059. Irisin+SB: 40 nmol/l r‐irisin with p38 inhibitor SB203580. Results are shown as means ± S.D.; *P < 0.05.

Treatment of CFs with r‐irisin markedly increased p38 and ERK activation over time (Fig. 8A and B). Western blotting analysis of samples treated for 10–60 min. revealed that activation peaked between 10 and 50 min., but started to decrease after 60‐min. treatment; the total protein amounts of p38 and ERK did not change during this period. Moreover, treatment with 0–40 nmol/l r‐irisin to examine the dose‐dependent effect revealed that 40 nmol/l r‐irisin significantly increased the phosphorylation of p38 and ERK without altering the p38 and ERK protein amounts (Fig. 8C and D). We further found that as compared with NG treatment, HG treatment slightly increased the activation of p38 and ERK, but HG exposure plus treatment with 40 nmol/l r‐irisin markedly increased p38 and ERK activation (Fig. 8E and F). Lastly, to further verify the role of p38 and ERK signalling pathways in r‐irisin/HG‐induced effects, CFs were pre‐treated with PBS added in normal medium (control; NG), r‐irisin in HG medium, the p38 inhibitor SB203580 (SB) plus r‐irisin in HG medium or the ERK inhibitor PD98059 (PD) plus r‐irisin in HG medium. The results showed that in CFs that were pre‐treated with SB or PD, the MMP‐2 and MMP‐9 up‐regulation induced by irisin and HG was significantly reduced (Fig. 8G and H).

## Discussion

Serum irisin levels were previously shown to be markedly higher in disease‐free centenarians than in patients with acute MI 18. Serum irisin level has also been demonstrated to be lower in patients with type 2 DM than in healthy controls 21, whereas elevated circulating irisin levels have been documented in patients with T1DM, particularly in female patients 17. Furthermore, irisin has recently been reported to lower blood glucose and induce insulin resistance in obese mice 15. However, none of the results from previous studies demonstrated the effects of irisin on DCM. Thus, we conducted both *in vitro* and *in vivo* studies to ascertain whether irisin influences the development of DCM.

In this study, we assessed the effects of r‐irisin treatment for 16 weeks after STZ‐induced development of T1DM in mice by investigating the changes in myocardial fibrosis, myocardial dysfunction, structural changes and DM‐related signalling pathways. In addition to using the mouse model of type I DCM, we used CFs or HUVECs to examine the influence of r‐irisin treatment. To determine whether r‐irisin can prevent the development of the characteristic changes of type I DCM, we used mice that were treated with r‐irisin to counteract the effects of DCM before the disease model was already established, and we used two doses of irisin (0.5 and 1.5 μg/g body weight/day) and continued the experiment for 8 weeks. We found that low‐dose irisin treatment substantially attenuated LV dysfunction and remodelling and decreased collagen deposition in diabetic mice *in vivo*, whereas the high dose of irisin did not exert a protective effect in diabetic mice. These results demonstrate that irisin produces a bidirectional effect on STZ‐induced diabetes in mice. Low‐dose irisin markedly reduced cardiac fibrosis *in vivo*, and irisin treatment *in vitro* inhibited HG‐induced EndMT by increasing Smad7 expression and suppressing the phosphorylation of Smad2 and Smad3 in HUVECs. The mechanisms underlying these positive effects of irisin thus may include inhibition of EndMT through TGF‐β/Smad signalling; this is because TGF‐β has been previously shown to potently induce the conversion of endothelial and epithelial cells into mesenchymal cells, and EndMT is characterized by the change in cell morphology to a spindle shape accompanied by a decrease in the expression of endothelial markers and an increase in the expression of mesenchymal markers 6, 22, 23. Here, we also showed that high‐dose irisin did not exert a marked beneficial effect, and the reason for this could be that high‐dose irisin up‐regulated the expression of MMPs in CFs, and such an increase in MMP expression through MAPK signalling might counteract the beneficial effect of inhibiting EndMT in HUVECs. This is the first study to characterize the effect of irisin in DCM and to investigate its potential roles in DCM. Our study further showed that the irisin effect on diabetic mice is dose‐dependent and bidirectional, and thus suggests that an optimal dose of irisin is required for treating DCM.

Another notable finding of this study is that irisin treatment reduced blood pressure in the DM group of mice. This agrees with the reported role of peripheral irisin in lowering blood pressure 24. This change might be related to the effect of irisin on the proliferation of vascular endothelial cells and suppression of HG‐induced apoptosis 25.

Diabetic cardiomyopathy is characterized by cardiac fibrosis and diastolic and systolic myocardial dysfunction coupled with high expression of inflammation‐associated cytokines such as TGF‐β1 26. We found that mice in the DM group frequently exhibited increased perivascular and intermyofibrillar fibrosis and dysfunction. Several *in vivo* and *in vitro* studies have demonstrated that chronic hyperglycaemia is a key initiating factor for multiple chronic diabetic complications 27. Endothelial cells exposed to hyperglycaemia start producing abnormal amounts of numerous proteins, and this altered production of proteins could result in the cellular phenotypic changes that are collectively referred to as EndMT. EndMT plays an essential role in the development of DCM and in the disease progression of other chronic diabetic complications and this, in turn, results in increased ECM protein production 28. EndMT has been reported to contribute substantially to cardiac fibrosis and heart failure 29. Recent studies have also implicated EndMT in the pathophysiology of vascular diseases including cerebral cavernous malformations, pulmonary hypertension, vascular graft remodelling and atherosclerosis 30, 31, 32, 33. Here, these cellular changes were observed *in vitro* in HUVECs after HG treatment. The results presented in Figure 8 not only show the diminished expression of endothelial markers in correspondence with the irisin dose, but also the effect of high‐dose irisin in the activation of CFs. As an essential factor in the induction of collagen production, HG could affect the transformation of endothelial cells into fibroblast‐like cells and thus stimulate collagen generation. Accordingly, our data showed that the expression levels of the mesenchymal markers FSP‐1, α‐SMA, collagen I and vimentin were elevated in HG‐treated HUVECs *in vitro*, whereas those of the endothelial markers CD31 and VE‐cadherin were decreased. We further demonstrated that both low‐dose and high‐dose irisin treatment regulated these processes. The production of both collagen I and collagen III was increased after high‐dose r‐irisin treatment *in vivo* (Fig. 6G). These results agree with the findings obtained with HUVECs, in which the HG‐induced changes were reversed by irisin treatment.

TGF‐β/Smad signalling exerts multifunctional effects on cellular metabolism, proliferation, migration, inflammation and fibrosis 34. TGF‐β functions as a key mediator in fibrosis that induces fibrotic diseases by activating downstream Smad signalling. Under certain disease conditions, Smad2 and Smad3 are highly activated, whereas Smad7 is degraded through the ubiquitin proteasome degradation mechanism 29, 34. As an inhibitory protein, Smad7, when overexpressed, inhibits the fibrotic effect by blocking Smad2 activation in TGF‐β‐induced renal tubular epithelial cells 9. All of these pathways are crucial for inducing EndMT, and hindering these pathways might disrupt the detrimental EndMT mechanism. In accordance with previous findings related to EndMT, we have shown here that irisin modulates cardiac EndMT through Smad‐mediated signalling. Irisin treatment also influenced the mechanisms related to EndMT development by increasing Smad7 expression and decreasing Smad2 and Smad3 phosphorylation.

In our study, both low‐ and high‐dose irisin targeting Smad signalling inhibited HG‐induced EndMT in HUVECs. However, high‐dose irisin did not produce a marked protective effect in mice with STZ‐induced diabetes. Complex mechanisms underlie the development of fibrotic diseases, and fibroblasts play a pivotal role in the development of the cardiac remodelling and fibrotic changes associated with DCM 35. Myocardial fibrosis plays a key role in the diabetic heart by regulating ECM deposition and turnover. MMPs are critical for the modification of ECM proteins, and, correspondingly, disruption of MMP activity results in excess collagen deposition 5. Although MMPs degrade ECM components, excessive MMP activation is concomitant with cardiac fibrosis in the rat diabetic myocardium 36. Conversely, inhibition of MMP activity was reported to prevent LV remodelling in a rabbit model 37. Recently, irisin was shown to promote MMP up‐regulation in HUVECs 38. Here, HG exposure up‐regulated MMP‐2 and MMP‐9 expression and irisin treatment strengthened this effect. However, under NG exposure, the irisin effect on CFs was not apparent. We suspect that certain unknown mechanisms are involved in the regulation of MMPs. For example, MAPK signalling pathways activated by hyperglycaemia contribute to the cardiovascular complications of diabetes 39. MAPKs play vital roles in cellular hypertrophy, cardiac cytokine‐mediated inflammation, cell proliferation and cardiac fibrosis 40. We found that in the heart of diabetic mice, high‐dose r‐irisin treatment increased ECM deposition (*i.e*. increased the myocardial expression of collagens I and III) as compared with the low‐dose treatment. Previously, p38 MAPK inhibition was shown to attenuate cardiac inflammation and prevent LV dysfunction in DCM 41, and ERK/MAPK signalling pathways were reported to function in the kidney fibrosis that was accelerated as a result of fluctuation in blood glucose levels 42. Thus, irisin might enhance and exacerbate collagen deposition by activating MAPKs. Our data here showed that r‐irisin treatment increased the level of phosphorylated p38 MAPK and ERK in mice with DCM.

In conclusion, this study addressed the function of irisin in DCM. Our findings have established, for the first time, a foundation for understanding the effect of irisin by identifying its potent effect on cardiac fibrosis in DM. Irisin has been widely reported to produce beneficial effects in diabetes; however, our results indicate that whereas low‐dose irisin alleviates cardiac fibrosis by inhibiting EndMT, high‐dose irisin enhances MMP expression thus likely MMP activity, which leads to abnormal synthesis and/or degradation of the ECM. Accordingly, low‐dose irisin markedly improved cardiac function in DM, but high‐dose irisin led to cardiac fibrosis and did not ameliorate diastolic and systolic myocardial dysfunction. Specifically, our results showed that high‐dose irisin treatment aggravated cardiac fibrosis and failed to alleviate diastolic and systolic LV dysfunction and cardiac remodelling *in vivo*, and induced MMP expression *in vitro*, which appears to be partly through the activation of p38 and ERK signalling. Identifying these signalling pathways involving irisin and EndMT in the pathology of DCM and cardiac fibrosis is crucial in clinical medicine. For example, pharmacological agents that restrict the progression of cardiac EndMT, which is observed in mice under pathological conditions, could be used in dosage‐specific irisin targeting, which might be helpful in preventing and treating cardiac fibrosis. Acquiring such knowledge could facilitate the design of therapeutic strategies for EndMT‐associated diseases such as fibrosis, cancer, diabetes and atherosclerosis 43.

## Conflict of interest

All authors declare that they have no conflict of interest.
